# Design of MEMS Gas Sensors and Integration for Multiple Gas Classification for Lithium-Ion Battery Thermal Runaway Warning

**DOI:** 10.3390/ma19112419

**Published:** 2026-06-05

**Authors:** Haiping Liu, Sen Zhang, Shan Xue, Delong Liu, Zeyu Sun, Lianshi Li, Qi Zhang, Mingzhi Jiao

**Affiliations:** 1 Beijing Institute of Mechanical Equipment, Beijing 100854, Chinaxues0414@163.com (S.X.); liudelong2016@163.com (D.L.); zeyu_sun96@163.com (Z.S.);; 2 CASIC Space Engineering Development Co., Ltd., Beijing 100080, China; 17778058937@163.com; 3 National & Local Joint Engineering Laboratory for Internet Application Technology in Mines, Xuzhou 221116, China

**Keywords:** MOS gas sensor, electronic noses, gas classification, micro-hotplate, ZnO, lithium-ion battery thermal runaway

## Abstract

Characteristic gas-based detection technology can facilitate the warning of lithium-ion battery thermal runaway with a high accuracy at an early stage. Microelectromechanical system (MEMS) metal–oxide–semiconductor (MOS) gas sensors have advantages of a low cost, a high accuracy, and low power consumption; therefore, they are ideal candidates for the lithium-ion battery thermal-runaway warning. MEMS MOS gas sensors are composed of a micro-hotplate and gas-sensitive materials. The micro-hotplate component strongly influences the device’s mechanical and thermal properties. Initially, we used COMSOL to optimize the micro-hotplate component. Then, we fabricated the device based on the optimal micro-hotplate. Next, gas-sensitive materials made of ZnO and ZnO-Au were deposited on the micro-hotplate by radio-frequency magnetic sputtering. The self-made and commercial MEMS MOS sensors were integrated to form an electronic nose. The as-made electronic nose can classify hydrogen, ethylene, acetylene, methane, carbon monoxide, and ethanol with a maximum accuracy of 99.4% using gas response data acquired over only 20 s. The reported work can provide a solution for an early and accurate lithium-ion battery thermal runaway warning.

## 1. Introduction

With the widespread application of lithium-ion batteries in electric vehicles, large-scale energy storage systems, and portable electronic devices, safety concerns have become a focal point for both academia and industry. Traditional battery management systems (BMSs) primarily rely on monitoring electrothermal parameters such as the voltage, current, and temperature to predict faults. However, these methods often trigger alarms only after irreversible side reactions (e.g., electrolyte decomposition, separator melting) reach critical levels, resulting in delayed warnings and a limited fault-localization accuracy [1,2,3,4]. In recent years, gas-detection-based early-warning strategies have demonstrated significant advantages. Under abuse conditions like overheating, overcharging, or internal short circuits, lithium-ion batteries decompose their electrolytes and electrode materials, releasing characteristic gases, including hydrogen (H_2_), carbon monoxide (CO), carbon dioxide (CO_2_), methane (CH_4_), and hydrocarbon gases such as ethylene (C_2_H_4_) and acetylene (C_2_H_2_) [5,6]. The six target gases selected in this study—H_2_, CO, CH_4_, C_2_H_4_, C_2_H_2_, and C_2_H_5_OH—are the most widely reported characteristic gases associated with lithium-ion battery TR across multiple battery chemistries. H_2_ and CO are produced by electrolyte reduction and oxidation reactions at early TR stages; CH_4_ and C_2_H_4_ arise from electrolyte decomposition at intermediate temperatures; C_2_H_2_ is a high-temperature marker appearing near cell venting; and C_2_H_5_OH is a solvent decomposition product. The literature reports that these gases appear at concentrations ranging from tens to thousands of ppm prior to catastrophic failure, which falls within the detection range of MOS sensors. Ethanol was included as a representative volatile organic compound that may co-exist in battery module environments. To achieve this goal, gas sensors, as core sensing components, play a pivotal role [7].

The current gas detection technologies encompass diverse approaches, including metal–oxide–semiconductor (MOS) sensors, electrochemical sensors, catalytic combustion sensors, and optical principle-based sensors. Among these, MOS gas sensors have been extensively studied for their advantages, including their low cost, high sensitivity, and long lifespan [8,9]. However, traditional bulk-type or thick-film sensors typically operate at high temperatures (300–500 °C), consume significant power, and face challenges in miniaturization, which limits their use in space-constrained and power-sensitive embedded systems (e.g., within battery modules). The emergence of microelectromechanical system (MEMS) technology has provided an ideal platform for revolutionary advancements in gas sensors. MEMS-manufactured micro-hotplate gas sensors integrate heating electrodes, insulating layers, and sensing materials onto micron-scale suspended membrane structures [10,11]. These architectures possess inherent advantages, including a low thermal capacity, a rapid thermal response, ultra-low power consumption (down to milliwatt levels), and ease of array integration, perfectly aligning with the IoT era’s urgent demands for miniaturized, low-power, and highly integrated smart sensing nodes. Current research progress in high-performance MEMS gas sensors primarily focuses on two complementary directions: the structural design and optimization of MEMS micro-hotplates to enhance their mechanical stability and thermal efficiency, and innovation in sensitive material modification to improve the sensitivity, selectivity, and stability for target gases.

At present, micro-hotplates are primarily classified into closed-membrane and suspended-membrane types. For the closed-membrane type, an inverted trapezoidal cavity is etched on the back of the substrate via anisotropic etching, and the top is hermetically sealed with a thin-film layer. In contrast, the suspended type is etched on the top, leaving the central heating region suspended by cantilevers. This structure results in a smaller heat-conduction area than the closed-membrane type, yet its mechanical strength is also reduced. Consequently, the two types of micro-hotplates exhibit distinct characteristics in structural stability and heating efficiency, with the suspended micro-hotplate achieving a lower power consumption than the closed-membrane type. In this paper, a suspended micro-hotplate was selected for the simulation analysis, given the size and power-consumption requirements of the IoT application. In micro-heating plate design, the material selection and geometric parameters (such as the film shape, thickness, and cantilever or bridge structures) of multilayer structures (including support membranes, heaters, insulation layers, and electrodes) directly determine the devices’ thermal and mechanical properties [12]. An optimized micro-heating plate structure can ensure uniform heating and a rapid thermal response while minimizing heat loss and maintaining structural stability and functional reliability during long-term operation, which is critical for sensor consistency and lifespan. For instance, optimizing thermal-insulation structures can significantly reduce the device power consumption—a decisive factor for battery-powered portable or wireless sensing systems [13]. Regarding sensitive materials, researchers continuously enhance sensors’ intrinsic performance through strategies such as nanostructure modulation, noble-metal catalysis, and heterostructure construction [11,14,15,16]. ZnO is one of the most investigated materials for gas sensing, owing to its n-type conductivity, defect diversity, and crystalline structure [17]. For instance, ZnO/Au/ZnO-based MEMS sensors have been reported to exhibit a high response to ethanol [18]. SnO_2_-Ag-ZnO thin-film-based MEMS sensors can detect methane with a strong response and very swiftly [19]. ZnO nano-bowls with Pd nanoparticles can also detect low concentrations of methane [20]. In addition, ZnO film can detect carbon monoxide very efficiently [21]. PdAu or Ce doping has been shown to enhance the hydrogen-sensing performance of ZnO [22,23]. Even though modifications can enhance ZnO’s selectivity for a specific gas, a single ZnO-based gas sensor still cannot detect mixed gases in a lithium battery scenario [24]. An electronic nose with multiple gas sensors and pattern-recognition algorithms can identify a gas in a mixed-gas background. Multiple layer perception was shown to achieve a very high accuracy in gas classification tasks and in hardware deployment [25,26].

The paper presents a systematic solution for the early and accurate recognition of thermal-runaway gases in lithium-ion batteries. “The objective of this study is threefold: (1) to optimize the structural parameters of a suspended MEMS micro-hotplate through a COMSOL finite-element simulation, focusing on the effects of the support layer material and thickness on temperature uniformity and mechanical deformation; (2) to fabricate and characterize ZnO- and Au-decorated ZnO gas-sensitive thin films on the optimized micro-hotplate; and (3) to evaluate the gas classification performance of a hybrid electronic nose—composed of both commercial and self-made MEMS MOS sensors—using a multilayer perceptron (MLP) algorithm for the six characteristic TR gases listed above.”

## 2. Materials and Methods

### 2.1. Simulation of MEMS Hotplate

1. Basic design

**Figure 1:**
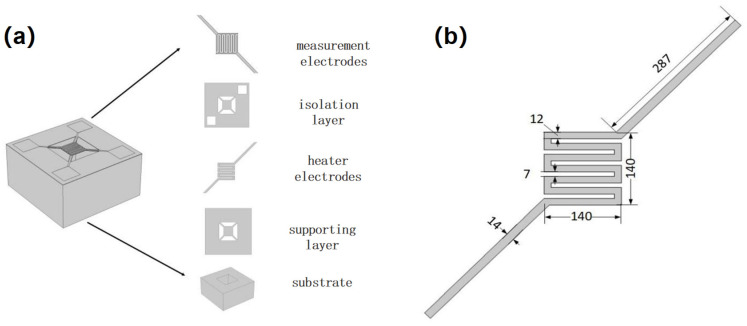
(**a**) Structure diagram of the MEMS sensors; (**b**) structure design of the serpentine micro-heater structure; unit: micrometer.

A suspended micro-hotplate can be supported by one or more cantilevers, with the four-cantilever configuration being the most widely used. Its structure, as shown in Figure 1a, comprises stacked functional layers, including, from bottom to top, a silicon substrate, a support layer, a heater electrode, an isolation layer, and a sensing electrode. A thermal-insulation cavity is formed in the silicon substrate via anisotropic etching. The support layer serves as the load-bearing structure of the micro-hotplate and is typically made of a single-layer silicon oxide or silicon nitride film, or a multilayer composite film, whose thickness significantly influences the micro-hotplate’s performance. The heater electrode is designed to maintain a stable operating temperature for the micro-hotplate and is typically serpentine in structure. The isolation layer separates the heater electrode from the sensing electrode, preventing current leakage from the heater to the sensing electrode and thereby affecting the final detection results. The sensing electrode detects changes in physical quantities; in micro-hotplate gas sensors, it is in direct contact with the gas-sensitive material to accurately measure changes in its resistance.

**Scheme 1:**
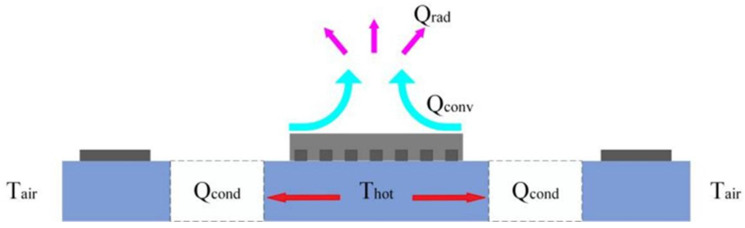
Schematic diagram of heat loss of the suspended micro-hotplate.

The heater electrodes of the micro-hotplate generate Joule heat under an electrical current; part of this heat is used to maintain the operating temperature of the gas-sensitive material, and the remainder is dissipated as thermal loss. The thermal loss of a micro-hotplate falls into three categories: heat conduction, heat convection, and heat radiation, as illustrated in Scheme 1. Among these, heat conduction is the dominant form, and the total thermal loss *Q_total_* can be expressed as:

*Q*_*total*_ = *Q*_*cond*_ + *Q*_*conv*_ + *Q*_*rad*_ + *x* (1)

where Q*_cond_*, Q*_conv_*, and Q*_rad_* denote heat conduction, heat convection, and heat radiation, respectively, and x represents the unaccounted thermal loss, including natural convection and other factors. Heat conduction is further classified into conduction between the micro-hotplate and air and between solid components. Since the thermal conductivity of air is much lower than that of the silicon substrate, only heat conduction between solid components is taken as the primary consideration.

The heat conduction through the cantilevers of the suspended micro-hotplate can be expressed as:

(2) $$Q_{cond}=\;G_{m}\lambda_{m}∆T_{l}=\;\frac{NA_{cond}}{l}\lambda_{m}∆T_{l}$$

where G*_m_* denotes the geometric factor of the micro-hotplate cantilevers, A*_cond_* the cross-sectional area, N the number of cantilevers, and l the length of a single cantilever. λ*_m_* represents the thermal conductivity of the film substrate with the unit of W·(m·K)^−1^, and ΔT_1_ is the temperature difference between the heating region and the boundary. Convective heat transfer Q*_conv_* refers to the heat exchange induced by the temperature difference when gas flows over the surface of the micro-hotplate, which can be expressed as:

*Q_conv_* = *A_conv_ h_f_* Δ*T*_2_ (3)

h_f_ denotes the gas convection coefficient with the unit of W·(m^2^·K)^−1^, Aconv is the convective heat transfer surface area, and ΔT_2_ represents the temperature difference between the micro-hotplate and the ambient gas environment. The micro-hotplate operates at an elevated temperature during operation and radiates energy to the surroundings; the magnitude of the radiated energy is dependent on the temperature and material properties of the micro-hotplate, which can be expressed as:

*Q_rad_* = *A_rad_ σε* (*T_h_*^4^ − *T_a_*^4^) (4)

A*rad* denotes the surface area for thermal radiation; σ is the emissivity, with a value ranging from 0 to 1; ε denotes the Boltzmann constant; Th is the temperature of the heating region of the micro-hotplate; and Ta represents the ambient boundary temperature. All temperatures involved in this experiment were below 700 °C, at which thermal radiation is negligible. Therefore, only heat conduction and heat convection were considered in the simulation model.

When the heating voltage is applied, the temperature distribution of the micro-hotplate can be obtained, and the displacement distribution is evaluated based on the thermal stress and mechanical static equilibrium equations:

σ = E(ε − εT)(5)

ε = αΔT(6)

where ε, εT, E, α, ΔT denote the strain, initial thermal strain, Young’s modulus matrix, coefficient of thermal expansion, and temperature difference, respectively.

Due to its hollow central structure, the selection of the support layer for the suspended micro-hotplate is particularly critical. The support layer must exhibit a high mechanical strength, an excellent thermal stability, and compatibility with standard semiconductor fabrication processes. Support layers are typically composed of either a single SiO_2_ layer or a composite layer of SiO_2_ and Si_3_N_4_. SiO_2_ was chosen as the single-layer support material over Si_3_N_4_ because the thermal conductivity of Si_3_N_4_ is significantly higher than that of SiO_2_, which would result in greater heat conduction to the silicon substrate and increased thermal loss. Compared with SiO_2_ films, Si_3_N_4_ films possess a higher hardness, a greater mechanical strength, and better chemical stability. Therefore, Si_3_N_4_ was used as the top and isolation-layer material in the composite support structure.

2. Simulation procedure of the MEMS hotplate

(1) Geometric Model Construction: The COMSOL Multiphysics 5.6 software was used for the finite-element analysis of the micro-hotplate to accurately simulate its temperature distribution, stress field, and deformation characteristics. The geometric model of the micro-hotplate was constructed at a 1:1 scale from the actual dimensions, consistent with those of a commercial micro-hotplate. The dimensions of the heating electrode are shown in Figure 1b: the overall size of the serpentine electrode area was 0.14 mm × 0.14 mm, the substrate size was 1 mm × 1 mm × 0.5 mm, and the central heating area was 0.16 mm × 0.16 mm. As planned, the thicknesses of the support and isolation layers were adjusted to examine the microplate’s temperature and stress distributions under varying thickness conditions.

(2) Material Selection: Different materials were selected for different layers of the micro-hotplate. The substrate was made of monocrystalline silicon. Silicon dioxide (SiO_2_) and silicon nitride (Si_3_N_4_) are commonly used as support and isolation layers in micro-hotplate manufacturing because they meet the required performance criteria. Platinum (Pt) was used as the heating and detection electrode due to its high thermal conductivity, low coefficient of thermal expansion, high melting point, and compatibility with standard silicon manufacturing processes. The specific material parameters used in the simulation are listed in Table 1.

(3) Setting of Physical Fields and Boundary Conditions: The simulation analysis of the micro-hotplate involved the coupling of three main physical fields: the electric current field, solid heat transfer field, and solid mechanics field. Corresponding boundary and domain conditions must be specified for each physical field to ensure the accuracy and reliability of the simulation results. In the solid heat transfer field, the initial temperature of the micro-hotplate and the temperature of the bottom boundary of the micro-hotplate were set to 25 °C, while the other boundaries were set to convective heat flux with a heat transfer coefficient of 20 W/(m^2^·K). In the electric current module of the multilayer shell, the heating voltage, ground connection, and electrical insulation parts were configured. In the solid mechanics module, the initial deformation was set to 0, and the bottom of the micro-hotplate was fixed.

(4) Setting of Physical Field Couplings: Electromagnetic–thermal coupling and thermal expansion coupling were added in the simulation. In addition, given the microplate’s thin-film structural characteristics, a solid thin-structure connection coupling was introduced.

(5) Mesh Generation: For the micro-hotplate, the focus was on observing the temperature and stress distribution in the central heating area and cantilever area. Therefore, dense meshes should be used in these key areas to ensure computational accuracy. The silicon substrate primarily serves as a support, and its thickness is much greater than that of the central area, with little impact on the simulation results. Thus, a relatively sparse mesh can be used for the silicon substrate. The mesh-setting scheme is shown in Figure S1.

(6) Calculation and Solution: A steady-state solver was selected because the objective was to observe the temperature and stress distribution of the micro-hotplate after it reached a stable state under multi-physics coupling. Because the thicknesses of the support and isolation layers varied, a parametric solver can more efficiently present the simulation results for the micro-hotplate across different parameter values.

### 2.2. Synthesis and Characterization of Sensing Materials for Self-Made MEMS Sensors

The sensing material for MEMS gas sensors was fabricated using a table-top sputtering machine. First, automatic vacuum pumping was started to reduce the pressure inside the chamber to below 5 × 10^−3^ Pa. Then, argon gas was introduced into the chamber at a constant flow rate of 30 sccm (standard cubic centimeter per minute) until the chamber pressure reached 1 Pa. Next, the sputtering power and time for the zinc oxide (ZnO) target were set as specified in Table 2; then, the radio-frequency (RF) power switch was turned on and the coating process was allowed to complete. After finishing the coating with the ZnO target, some micro-hotplates were taken out for reference. Then, the target material was switched to gold (Au). The chamber was re-evacuated to a pressure below 5 × 10^−3^ Pa; then, argon was introduced at a flow rate of 30 sccm until the pressure reached 1 Pa. The sputtering power and time were set for the gold target, the parameters were confirmed, and the RF switch was turned on. Once the thin-film preparation was complete, the RF switch, the gas-flow inlet, and the turbomolecular pump were turned off. When the turbomolecular pump frequency reached 0, the mechanical pump, gate valve, and power supply were turned off. The chamber was opened, and the samples were removed and labeled appropriately. The aging experiment was conducted in a KSL-1100 precision box furnace manufactured by Hefei Kejing Co. (Hefei, China). The heating temperature was set to 260 °C with a holding time of 12 h. This heat-treatment process helped eliminate residual stress within the material and improve interfacial contact, thereby enhancing the sensor’s long-term operational stability.

### 2.3. Gas Response Test and Algorithm Training

In this experiment, the static gas testing method was adopted. A total of 6 gases were selected: hydrogen, acetylene, ethylene, methane, carbon monoxide, and ethanol, with each gas assigned to a separate gas cylinder. To construct a comprehensive dataset, four distinct concentrations were set for each gas, and 15 samples were collected at each concentration. Thus, 60 samples were obtained for each gas, yielding a total of 360 samples. The specific experimental procedure for data acquisition was as follows: (1) Gas preparation: Extract each gas from the corresponding cylinder into a gas bag and label it properly. Place the test system hardware in the 1 L test chamber, which is equipped with a gas injection channel. After connecting the power supply, seal the interfaces with liquid sealant and leave the setup for at least 1 week to avoid interference from volatile gases during testing. Set the heating voltage of the self-developed sensor and its load resistance using a sliding rheostat. Open the host computer software and configure the corresponding parameters. (2) Gas injection and outgas: After accurately calculating the volume of gas to be injected each time, extract the gas from the gas bag and inject it into the test chamber. Then observe real-time changes in the signal waveform using the host computer software. Once the gas sensor response signal stabilizes, open the test chamber lid to initiate the sensor’s recovery phase. The entire acquisition process lasts 200 s. Upon completion, save the response data collected in this trial and complete the corresponding labeling. (3) Dataset construction: Repeat Step 2 until all experiments are finished. Finally, a self-developed dataset comprising 6 gases and 360 samples was constructed. The specific concentrations corresponding to each gas are detailed in Table 3. No explicit fitting of rise/fall time constants was performed; rather, the raw response time-series values were used directly as features. All the experiments were conducted with single-gas samples; the electronic nose was designed and validated to identify the dominant gas present. Extension to gas mixture classification is an important future direction.

## 3. Results and Discussion

### 3.1. Simulation Results of MEMS Micro-Hotplate

(1) Influence of SiO_2_ support layer on micro-hotplate

**Figure 2:**
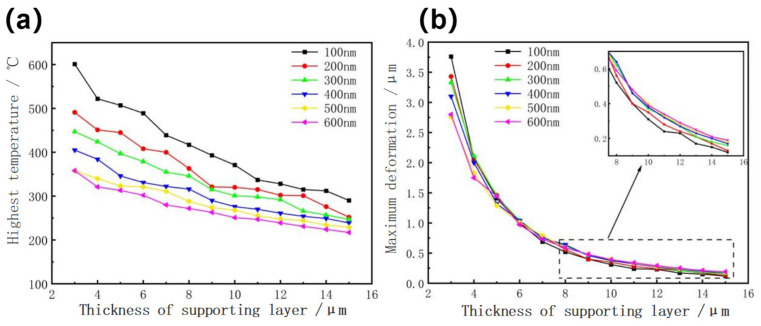
The influence of the thickness of the SiO_2_ support layer on the maximum temperature and deformation of the micro hotplate: (**a**) the maximum temperature; (**b**) the maximum deformation. Different colors refer to different thicknesses of the Si_3_N_4_ isolation layer.

The influence of the thickness of the single SiO_2_ support layer on the micro-hotplate was first investigated. In the experiments, the isolation layer thickness was fixed for each group, while the support layer thickness was varied from 3 µm to 15 µm at a heating power of 24.2 mW. The maximum temperature and deformation of the micro-hotplate were observed. A total of six experimental groups were conducted, with the isolation layer thickness ranging from 0.1 µm to 0.6 µm, as shown in Figure 2. It can be seen that, at the same isolation-layer thickness, the maximum temperature of the micro-hotplate decreased as the support-layer thickness increased, although the relationship between the support-layer thickness and temperature was non-linear. At the same support-layer thickness, a thicker isolation layer yielded a lower maximum micro-hotplate temperature, and the rate of temperature reduction with increasing support-layer thickness decreased. When the isolation layer thickness was held constant, the support layer thickness significantly affected the micro-hotplate’s deformation. As the support layer thickness increased, the micro-hotplate’s deformation gradually decreased and stabilized. When the micro-hotplate deformation stabilized, under the same conditions, the micro-hotplate with an isolation layer thickness of 0.1 μm reached the highest temperature among the groups. This indicates that, to reach the sensor’s operating temperature, the micro-hotplate with a 0.1 μm isolation layer required the least heating power, resulting in the lowest power consumption. However, this does not mean that the thinner the isolation layer, the better. An excessively thin isolation layer can cause electrical contact between the heating and sensing layers, leading to abnormal operation or sensor failure. An excessively thin isolation layer risks electrical contact between the heating and sensing layers, as is well established in micro-hotplate fabrication [12]; a minimum thickness of approximately 0.1 μm was therefore maintained in this study.

(2) Influence of SiO_2_ and Si_3_N_4_ composite support layer on micro-hotplate

**Figure 3:**
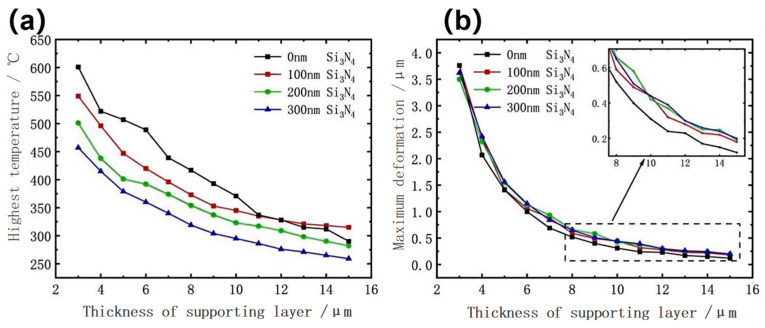
The influence of the thickness of different composite layer materials on the micro-hotplate: (**a**) the maximum temperature; (**b**) the maximum deformation.

Although Si_3_N_4_ has a higher thermal conductivity and a larger thermal expansion coefficient than SiO_2_, which leads to greater heat loss to the silicon substrate and larger deformation, it results in a more uniform temperature distribution in the central heating region. In the next experiment, the thickness of the isolation layer was fixed at 0.1 μm. The temperature and deformation of the micro-hotplate were investigated under different support layer thicknesses, with the top layer of the composite support being Si_3_N_4_ at 0.1 μm, 0.2 μm, and 0.3 μm. As shown in Figure 3, the overall trends in the temperature and displacement distributions were similar to those of the single-material support layer. However, at the same isolation layer thickness, the maximum temperature was lower than that of the micro-hotplate with a single-material support layer, while the temperature distribution curve of the micro-hotplate with a composite support layer was smoother. Under the same conditions, the thicker the Si_3_N_4_ layer, the lower the micro-hotplate’s maximum temperature.

As shown in Figure 3b, when the Si_3_N_4_ thickness was 0.1 μm and the total support layer thickness was 9 μm, the maximum deformation was 0.49 μm, representing a reduction of approximately 18% compared to the single-SiO_2_ support layer at the same maximum temperature (Figure 2b). Furthermore, as quantified by the COMSOL temperature distribution maps (see Figure 3a inset), the temperature difference in the central heating region decreased from ~28 °C (single SiO_2_) to within 10 °C (composite), confirming an improved heating uniformity.

Adding the Si_3_N_4_ layer improved the temperature uniformity in the micro-hotplate’s central region. With a composite support layer of 0.1 μm Si_3_N_4_, the temperature difference in the central region of the micro-hotplate was within 10 °C, thus improving the heating uniformity. Here, the “central region” is defined as the inner 60% of the membrane area (approximately 0.096 mm × 0.096 mm within the 0.16 mm × 0.16 mm heating area). For the single-material SiO_2_ support at the same total thickness and same maximum temperature, the COMSOL simulation yielded a temperature difference of approximately 28 °C across the central region. With the 0.1 µm Si_3_N_4_ composite support, the temperature difference in the same region reduced to within 10 °C—a ~64% improvement in heating uniformity.

### 3.2. Gas Sensor Characterization

**Figure 4:**
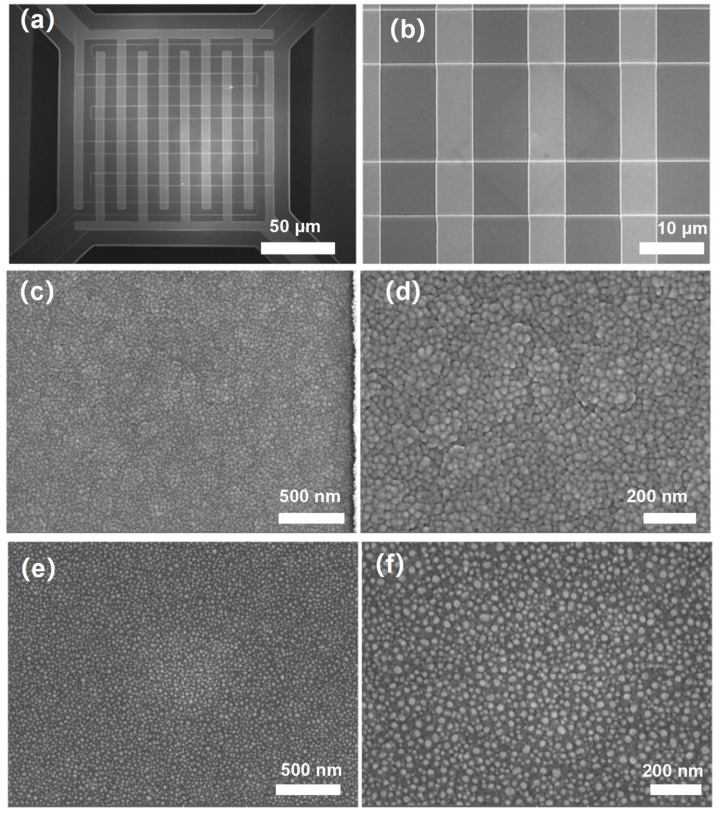
SEM images of the self-made sensor chips with different sensing materials: (**a**–**d**) ZnO; (**e**,**f**) Au-ZnO.

The pure ZnO gas-sensitive thin film exhibited a dense and highly ordered surface structure. In contrast, the Au-modified ZnO material was decorated with numerous distinct spherical nanostructures on the surface (Figure 4). These unique morphological features significantly increase the contact area between the material and gas molecules, providing more active sites for gas adsorption and reaction, thereby effectively improving the sensor’s detection sensitivity and response speed.

**Figure 5:**
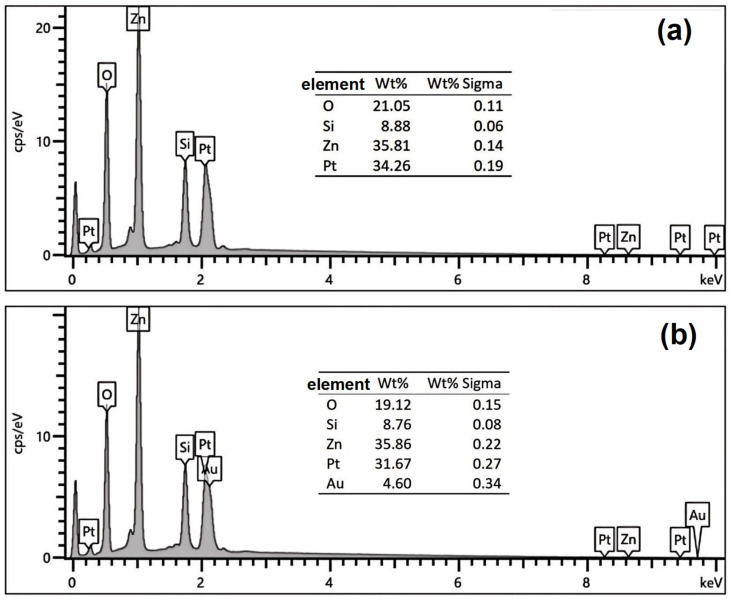
EDS diagram of different sputtered ZnO samples: (**a**) ZnO; (**b**) ZnO-Au.

According to the energy-dispersive X-ray spectroscopy (EDS) results, clear characteristic peaks for Zn and O were observed, indicating the successful preparation of ZnO. Meanwhile, distinct characteristic Au peaks were detected in the EDS spectrum of Au-doped ZnO, confirming that Au elements were successfully incorporated into the ZnO material, as seen in Figure 5. ZnO has a work function of 3.9 eV, while Au has a work function of 5.1 eV [27]. As a result, the electrons will move from the conduction band of ZnO toward Au, thus forming a Schottky contact. Therefore, the depletion layer in ZnO will be wider, thereby enhancing the sensitivity to reductive gases. EDS mapping showed that Au was uniformly decorated on the ZnO surface, as shown in Figure S4.

**Figure 6:**
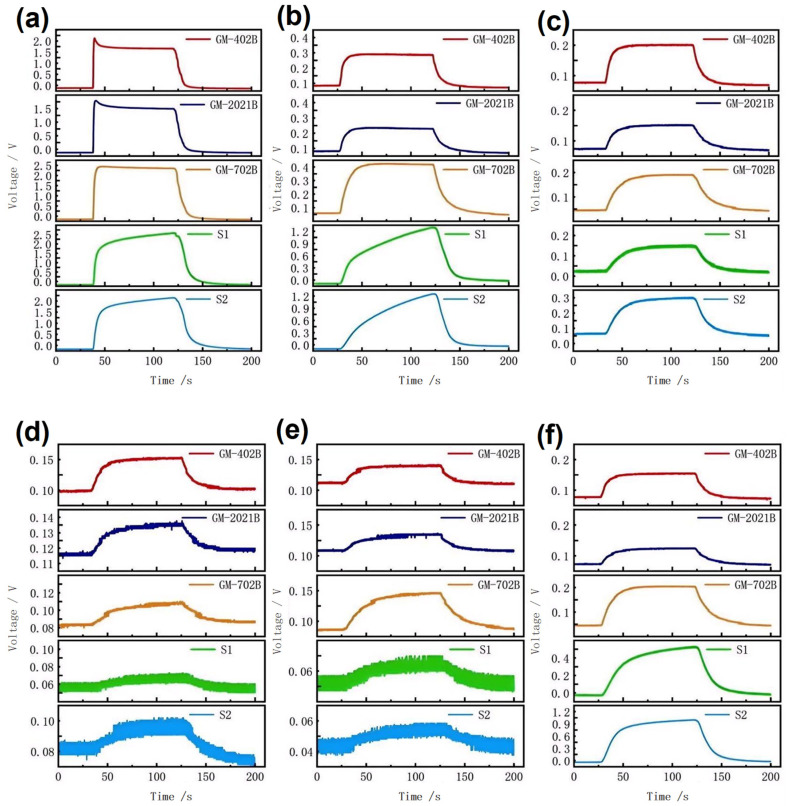
The dynamic responses of the five sensors toward different gases: (**a**) 400 ppm hydrogen; (**b**) 250 ppm acetylene; (**c**) 800 ppm ethylene; (**d**) 4000 ppm methane; (**e**) 40 ppm CO; and (**f**) 100 ppm ethanol.

As seen in Figure 6, the dynamic responses of the five sensors to the six gases differed in their response value, response time, and recovery time. These varieties made discrimination of the six types of gases achievable by using the gas sensor arrays. The repeatability of the measurements was also very high, as shown in Figure S5, showing a very good precision for the test.

**Figure 7:**
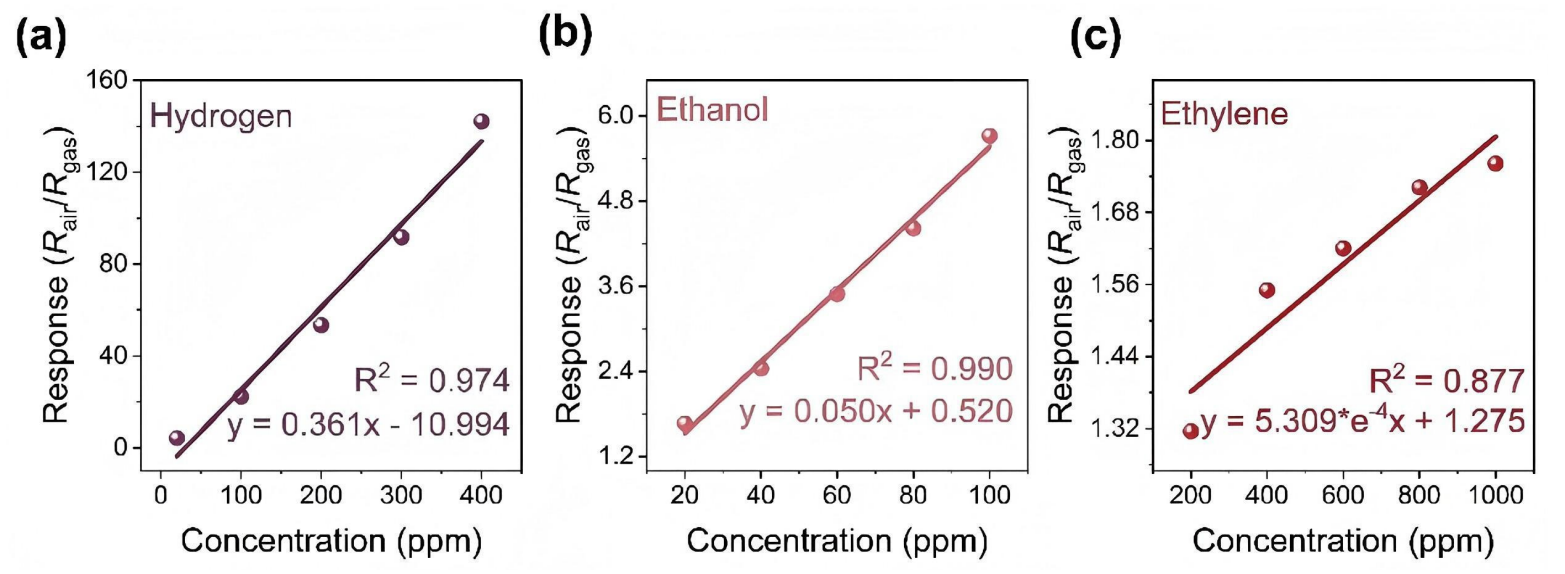
Linear fitting curves of response versus gas concentration for three different gases: (**a**) hydrogen, (**b**) ethanol, and (**c**) ethylene. The fitted equations and R^2^ values are labeled in the plots; the limits of detection were calculated using LOD = 3*sigma/slope.

The response values at different concentrations were fitted linearly for hydrogen, ethanol, and ethylene, as shown in Figure 7. The LOD was calculated using LOD = 3*sigma/slope, where sigma is the standard deviation of the baseline noise and the slope is obtained from the calibration curve. The calculated LOD values were 37.7 ppb for hydrogen, 255.1 ppb for ethanol, and 26.2 ppm for ethylene.

We also summarized the response value, response time, and recovery time at each gas concentration in Table 4. These additional data provide a clearer comparison of the sensing performance, including the sensitivity, detection capability, and dynamic response. An array composed of different sensing units would be valuable for improving gas discrimination and the fitting accuracy. The present work focuses on the LOD and dynamic response evaluation of the current-sensing material, and the construction of a multi-sensor array will be considered in the next stage of this study.

### 3.3. Results of MLP Algorithms

**Figure 8:**
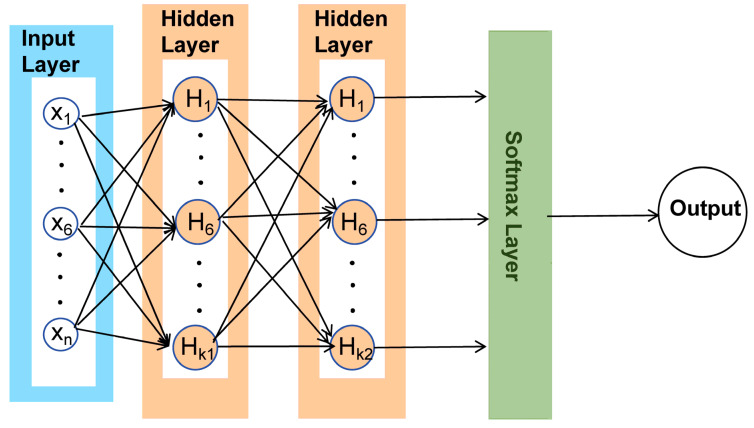
Structure of the four-layer multilayer perceptron (MLP) architecture.

In this study, a four-layer multilayer perceptron (MLP) architecture, as illustrated in Figure 8, was adopted, consisting of one input layer, two hidden layers, and one output layer. To prevent overfitting, a dropout layer with a dropout probability of 0.2 was added after each hidden layer. The number of nodes in the input layer was determined by the dimensionality of the sample features, while the number of nodes in the output layer corresponded to the number of classification categories. The number of neurons in the hidden layers was optimized via a grid search over hyperparameters, with each layer’s neuron count constrained to be less than 25.

The model was trained using the TensorFlow deep learning framework to accurately classify six gases: methane, hydrogen, carbon monoxide, ethanol, acetylene, and ethylene. For this classification task, sparse categorical cross-entropy was used as the loss function, and adaptive moment estimation (Adam) as the optimization algorithm. The ReLU activation function was used for the hidden layers, which offers advantages such as a high computational efficiency, mitigation of the vanishing gradient problem, and accelerated model convergence. The Softmax activation function was applied to the output layer to convert the model’s raw outputs into a probability distribution, thereby meeting the requirements of the multi-classification task. During training, the batch size was set to 32 and the learning rate to 0.02. The aforementioned parameter settings and hyperparameter optimization were intended to construct a gas classification model suitable for the system in this study.

### 3.4. Influence of Data Length on Classification Results

**Figure 9:**
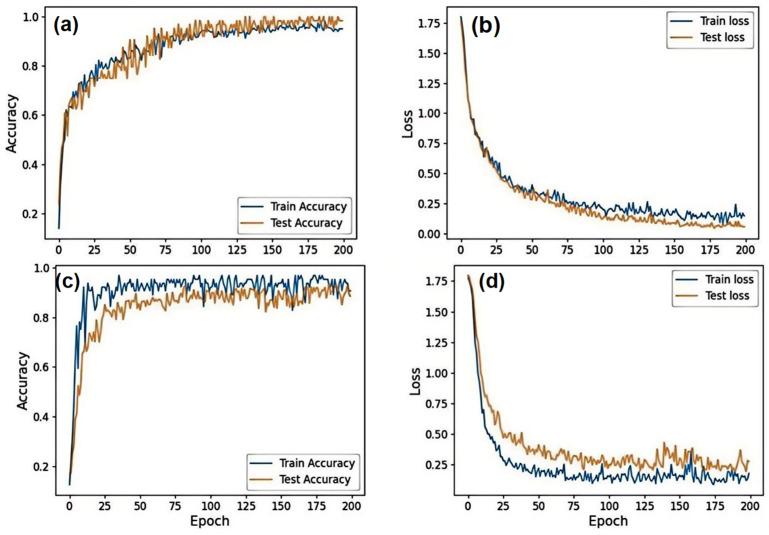
Experimental results of the 5 s response data of different components of electronic noses: (**a**,**b**) three commercial and two self-made sensors; (**c**,**d**) three commercial sensors.

As shown in Figure 9a,b, when using five sensors and combining commercial and self-made sensors, the accuracy and loss curves stabilized after 150 epochs with 5 s response data. Using only commercial sensors, the accuracy and loss curves stabilized after 75 epochs with 5 s response data.

**Figure 10:**
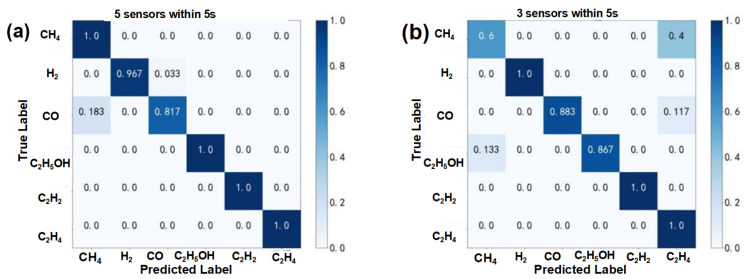
Comparative analysis of confusion matrices for gas identification by different gas sensor arrays within a 5 s response time window: (**a**) three commercial and two self-made sensors; (**b**) three commercial sensors.

Using a 5 s response time window, a gas sensor array composed of five sensors can perform well in most cases, except that H_2_ may be misclassified as CO with a probability of 0.33 and CO may be misclassified as CH_4_ with a probability of 0.183, as shown in Figure 9a. Using a 5 s response time window, a gas sensor array composed of only three commercial sensors can perform well in most cases, except that CH_4_ may be misclassified as C_2_H_4_ with a probability of 0.33 and CO may be misclassified as C_2_H_4_ with a probability of 0.117, as shown in Figure 10b.

**Figure 11:**
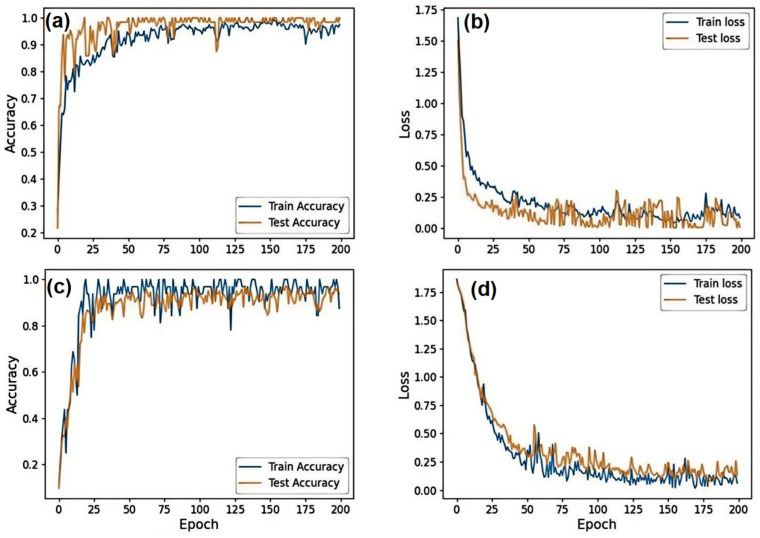
Experimental results of the 10 s response data of different components of electronic noses: (**a**,**b**) three commercial and two self-made sensors; (**c**,**d**) three commercial sensors.

As shown in Figure 11a,b, when using five sensors and combining commercial and self-made sensors, the accuracy and loss curves stabilized after 60 epochs with 10 s response data. Using only commercial sensors, the accuracy and loss curves stabilized after 125 epochs with 100 s of response data.

**Figure 12:**
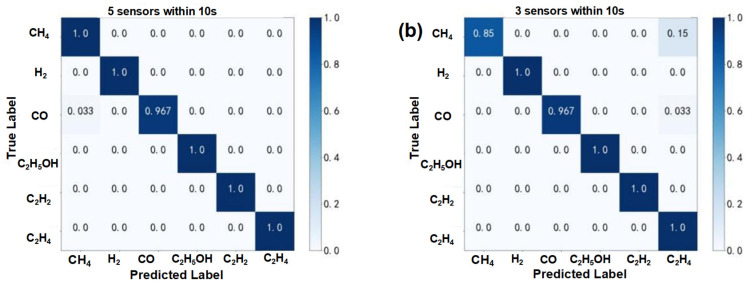
Comparative analysis of confusion matrices for gas identification by different gas sensor arrays within a 10 s response time window: (**a**) three commercial and two self-made sensors; (**b**) three commercial sensors.

Using a 10 s response time window, a gas sensor array composed of five sensors can perform well in most cases, except that CO may be misclassified as CH_4_ with a probability of 0.033, as shown in Figure 11a. When using a 10 s response time window, a gas sensor array composed of only three commercial sensors can perform well in most cases, except that CH_4_ may be misclassified as C_2_H_4_ with a probability of 0.15 and CO may be misclassified as C_2_H_4_ with a probability of 0.033; see Figure 12b.

**Figure 13:**
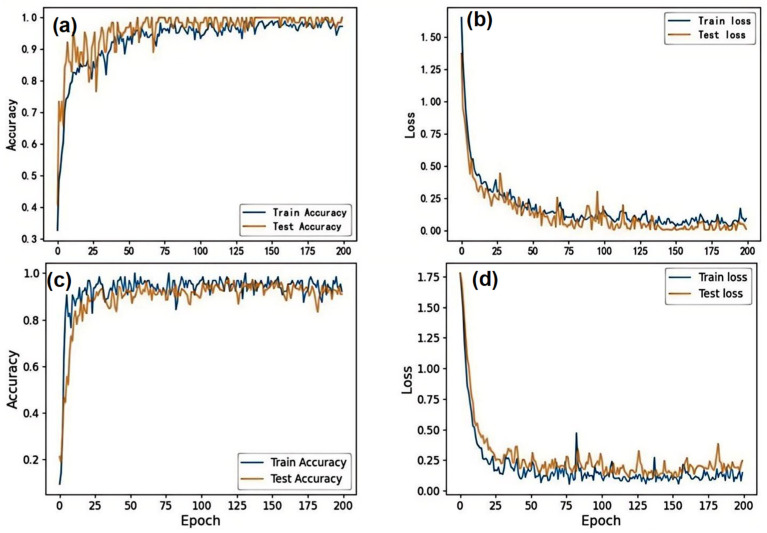
Experimental results of the 15 s response data of different components of electronic noses: (**a**,**b**) three commercial and two self-made sensors; (**c**,**d**) three commercial sensors.

As shown in Figure 13a,b, when using five sensors and combining commercial and self-made sensors, the accuracy and loss curves stabilized after 150 epochs with 15 s response data. Using only commercial sensors, the accuracy and loss curves stabilized after 120 epochs with 15 s response data.

**Figure 14:**
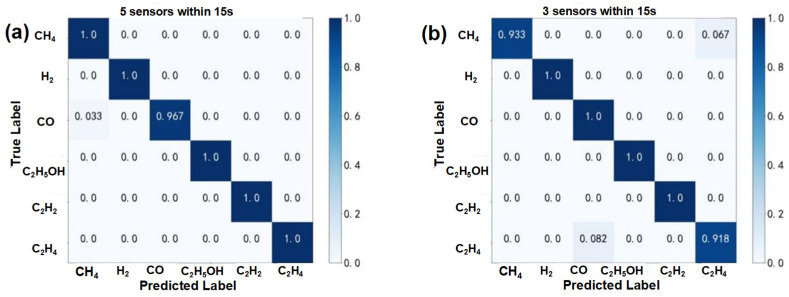
Comparative analysis of confusion matrices for gas identification by different gas sensor arrays within a 15 s response time window: (**a**) three commercial and two self-made sensors; (**b**) three commercial sensors.

Using a 15 s response time window, a gas sensor array composed of five sensors can perform well in most cases, except that CO may be misclassified as CH_4_ with a probability of 0.033, as shown in Figure 14a. Using a 15 s response time window, a gas sensor array composed of only three commercial sensors can perform well in most cases, except that CH_4_ may be misclassified as C_2_H_4_ with a probability of 0.067 and C_2_H_4_ may be misclassified as CO with a probability of 0.082; see Figure 14.

**Figure 15:**
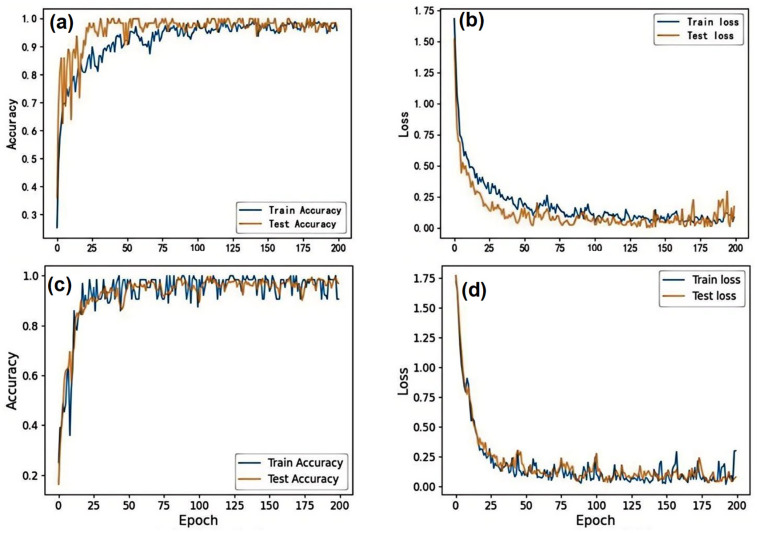
Experimental results of the 20 s response data of different components of electronic noses: (**a**,**b**) three commercial and two self-made sensors; (**c**,**d**) three commercial sensors.

When using five sensors and combining commercial and self-made sensors, the accuracy and loss curves stabilized after 125 epochs with 20 s response data (Figure 15a,b). Using commercial sensors, the accuracy and loss curves stabilized after 110 epochs with 20 s response data (Figure 15c,d).

**Figure 16:**
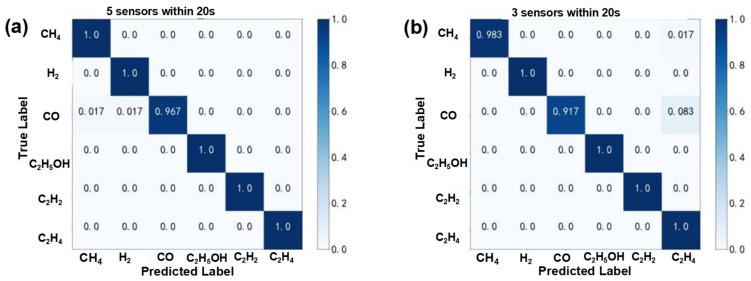
Comparative analysis of confusion matrices for gas identification by different gas sensor arrays within a 20 s response time window: (**a**) three commercial and two self-made sensors; (**b**) three commercial sensors.

Using a 20 s response time window, a gas sensor array composed of five sensors can perform well in most cases, except that CO may be misclassified as H_2_ or CH_4_ with probabilities of 0.017 and 0.017, respectively, as shown in Figure 13a. Using a 20 s response time window, a gas sensor array composed of only three commercial sensors can perform well in most cases, except that CH_4_ may be misclassified as C_2_H_4_ with a probability of 0.017 and CO may be misclassified as C_2_H_4_ with a probability of 0.083, as shown in Figure 16b.

As shown in Table 5, the combination of commercial sensors and two self-made sensors also showed a higher accuracy than bare commercial MEMS sensors. MEMS gas sensor arrays composed of five sensors can achieve a 99.2% classification accuracy in just 20 s. This is higher than our work when using only three commercial gas sensors. In addition, our results with five sensors were similar to those in another work using three commercial MEMS MOS sensors [28]. We primarily investigated the fundamental concepts of the measurement system for lithium battery thermal-runaway warnings. The ESP32 MCU was employed in the present work. It generally has a relatively large size and a medium power consumption. However, for real-world applications, some smaller, low-power MCUs can be chosen. The measurement system can be placed at the edge of the real battery pack cases. Due to the easy dissipation of gases in the battery pack cases, the placement of the gas sensor arrays will not affect the timeliness of the warning as much as temperature or pressure sensors.

## 4. Conclusions

Here, we provided a good solution for thermal-runaway warnings of lithium-ion batteries by accurate characteristic-gas recognition using MEMS MOS gas sensor arrays and pattern-recognition algorithms. The microplate for the MEMS MOS gas sensor was first simulated. The optimal component of the isolation layer was determined by considering both power consumption and thermal stability. Then, based on the optimized micro-hotplate, the sensing materials, the ZnO and ZnO-Au films, were deposited using a magnetron sputtering machine. Au decoration enhanced the sensitivity of the ZnO-Au gas sensors through the Schottky barrier effect. A gas sensor array was formed by combining commercial gas sensors with self-made sensors integrated with PCBs and ESP32 MCUs. With the help of an MLP, the gas sensor arrays composed of three commercial and two self-made sensors could discriminate six single gases with a 98.5% accuracy within 10 s, showing great potential for further applications in the early warning of lithium-ion battery thermal runaway.

However, our work has some limitations: long-term mechanics and drift need to be investigated later, since these issues may greatly affect the accuracy. A real lithium-battery thermal-runaway scenario test should be carried out to demonstrate the technique’s actual performance. The work still provides a laboratory-scale demonstration rather than verification of real-world applications.

## Figures and Tables

**Table 1:** Material parameters of the micro-hotplate simulation model.

Materials	Si	Pt	SiO_2_	Si_3_N_4_
Resistivity (Ω·m)	1 × 10^−3^	1.05 × 10^−7^	1 × 10^12^	1 × 10^12^
Thermal conductivity (W/(m·K))	130	71.6	1.4	20
Heat capacity (J/(kg·K))	700	133	730	700
Thermal expansion Coefficient (10^−6^/K)	2.6	8.8	0.5	2.3
Density (kg/m^3^)	2329	21,450	2200	3100
Young’s modulus (GPa)	170	168	70	250

**Table 2:** Sputtering parameters of different target materials.

Target	Sputtering Time/s	Ar Flow Rate /sccm	RF Power /W	Thickness /nm
ZnO	1800	30	50	192
Au	20	30	50	5

**Table 3:** Information of test concentration for each gas.

	Hydrogen /ppm	C_2_H_2_ /ppm	C_2_H_4_ /ppm	CH_4_ /ppm	CO /ppm	C_2_H_5_OH /ppm
Test concentration	100	100	200	1000	25	40
	200	150	400	2000	30	60
	300	200	600	3000	35	80
	400	250	800	4000	40	100
Number of samples	60	60	60	60	60	60

**Table 4:** Response values, response times, and recovery times were extracted from the dynamic sensing curves for hydrogen, ethanol, and ethylene at different concentrations.

Gas	Concentration (ppm)	Response Value	Response Time (s)	Recovery Time (s)
Hydrogen	20	4.182	28	13
	100	22.273	72	8
	200	53.323	92	6
	300	91.632	96	6
	400	142.118	104	4
Ethanol	100	5.718	211	23
	80	4.414	191	25
	60	3.487	190	27
	40	2.439	144	29
	20	1.664	84	39
Ethylene	200	1.316	52	19
	400	1.551	50	43
	600	1.62	46	31
	800	1.722	47	37
	1000	1.761	50	29

**Table 5:** Comparison of the gas classification accuracy of the gas sensor arrays with different components and other works.

Gas Sensor Array Type	Time Window (s)	Classification Task	Accuracy (%)	Reference
Three commercial MEMS MOS sensors	20	2 single and 1 binary gas	99.2	[28]
Three commercial MEMS MOS sensors	10	6 single gases	96.8	This work
Three commercial plus two self-made MEMS MOS sensors	10	6 single gases	99.2	This work

## Data Availability

The original contributions presented in this study are included in the article/supplementary material. Further inquiries can be directed to the corresponding authors.

## Supplementary Materials

The following supporting information can be downloaded at: https://www.mdpi.com/article/10.3390/ma19112419/s1.
