# Supplementary material for: Design of MEMS Gas Sensors and Integration for Multiple Gas Classification for Lithium-Ion Battery Thermal Runaway Warning
Source: Materials (Basel). 2026 Jun 5;19(11):2419. doi: 10.3390/ma19112419 (PMC13258333; doi:10.3390/ma19112419)
Supplement: Supplementary file 1 [file materials-19-02419-s001.zip › materials-4270594-supplementary.pdf]

Design of MEMS gas sensors and integration for multiple gas classification for lithium-ion battery thermal runaway warning

Haiping Liu[1], Sen Zhang[1], Shan Xue[1], Delong Liu[1], Zeyu Sun[1], Lianshi Li[1], Qi Zhang[2], Mingzhi Jiao[3]

[1] Beijing Machinery Equipment Research Institute, Beijing 100854, China
[2.] CASIC Space Engineering Development Co., Ltd, Beijing 100080, China
[3] National & Local Joint Engineering Laboratory for Internet Application Technology in Mines, Xuzhou 221116, China

* Correspondence: Sen Zhang, zhangsen817@163.com
Mingzhi Jiao，mingzhijiao@cumt.edu.cn;

1. Scheme of mesh setting

Finite-element calculations rely on meshes, and the mesh division density is closely correlated with the accuracy of the final simulation results. Denser meshes lead to more accurate simulation outcomes, yet they also bring about an increased computational load and storage space consumption. For micro-hotplates, the temperature and stress distributions in the central heating zone and cantilever region are the main research focuses. Hence, fine meshes should be arranged in these key areas to guarantee the calculation accuracy. As the silicon substrate mainly serves a supporting function with a thickness far greater than that of the central region, it exerts a relatively minor influence on the simulation results and can be divided with relatively coarse meshes. Such a differentiated mesh configuration not only improves the computational efficiency, but also ensures the simulation precision of key regions. The mesh division results are presented in Fig.S1.

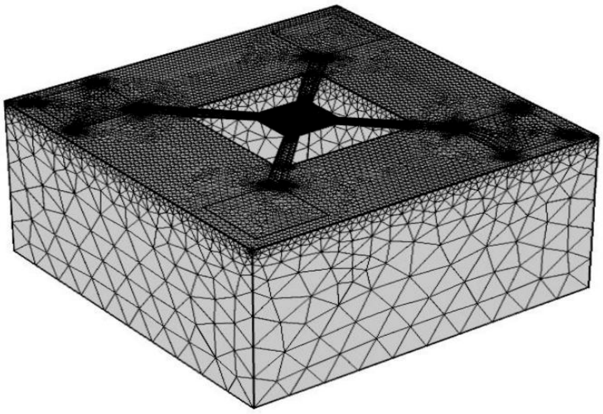

Fig.S1. Schematic diagram of the meshing of the micro-hotplate.

2. The component gas sensor array

As shown in Table S1, three commercial MEMS MOS gas sensors were used for the gas sensor array. To form a highly efficient gas sensor array, the two self-made MEMS MOS gas sensors were combined together with these three sensors.

Table S1. Information of the three commercial MEMS sensors for the gas sensor array.

Gas sensor type	Manufacturer	Target gases
GM-402B	Winsen Electronics	H2/CO/CH_4
GM-2021B	Winsen Electronics	VOCs, including ethanol
GM-702B	Winsen Electronics	CO/CH_4/LPG

A hybrid sensor array combining commercial and self-fabricated sensors was adopted to integrate the high stability, mature calibration, and strong anti-interference ability of commercial sensors with the high sensitivity, excellent specificity, and customizable materials of self-fabricated sensors. This scheme compensates for the poor consistency and long-term drift of purely self-fabricated sensor arrays, enriches the gas-responsive feature dimensions, and improves the recognition accuracy for mixed gases as well as the system practicability. The gas sensor array system is shown in Fig. S2.

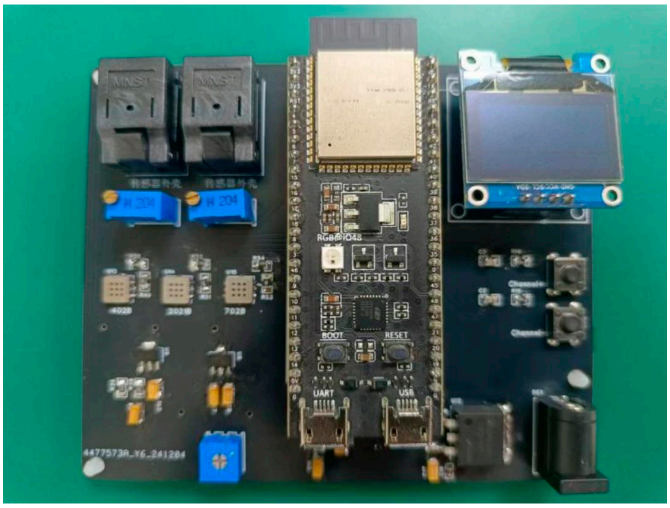

Fig.S2. Schematic diagram of the gas sensor array system with 3 commercial MEMS MOS gas sensors and 2 self-made MEMS MOS gas sensors in the black socket.

3. EDS mapping results of ZnO and ZnO-Au.

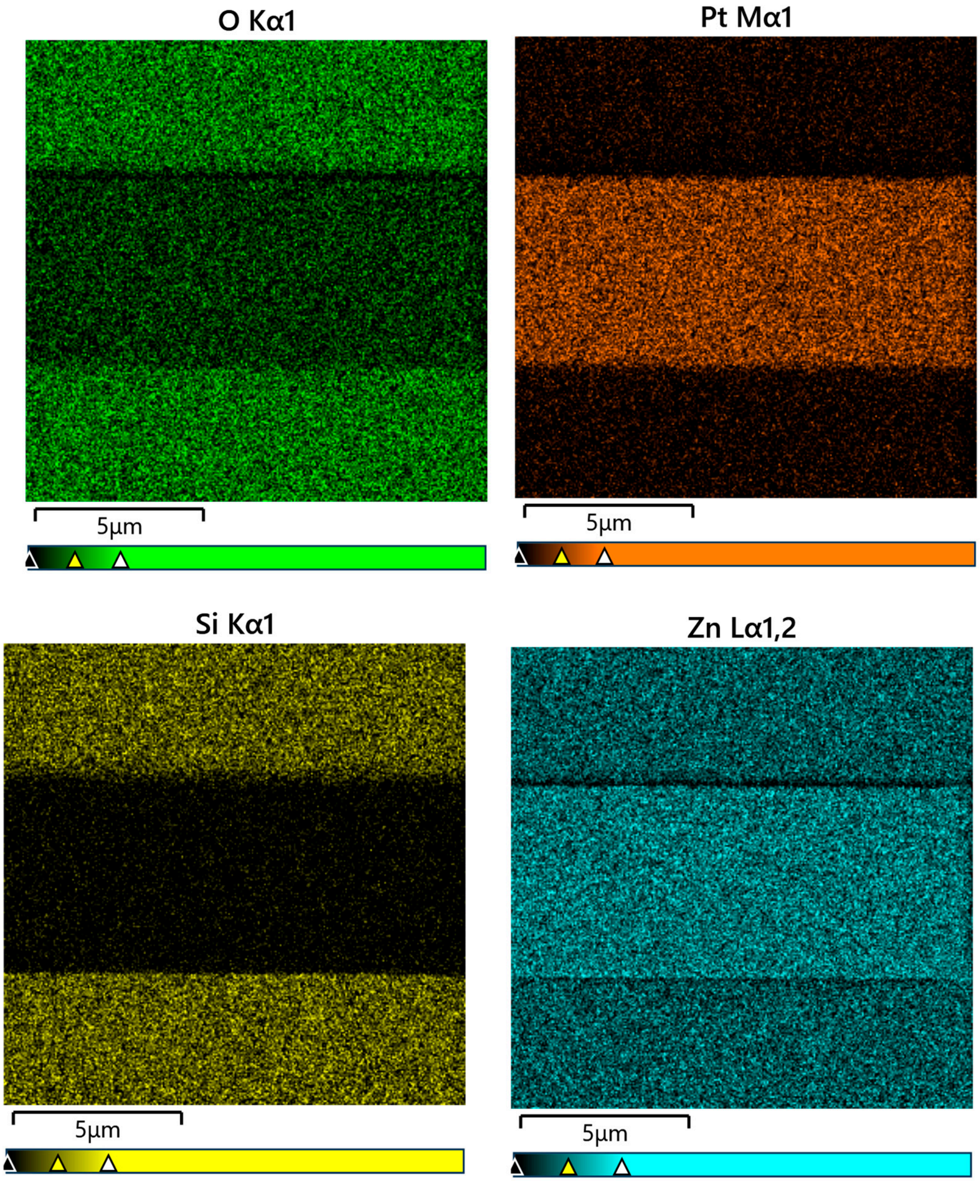

Fig. S3. EDS mapping of ZnO sensing materials.

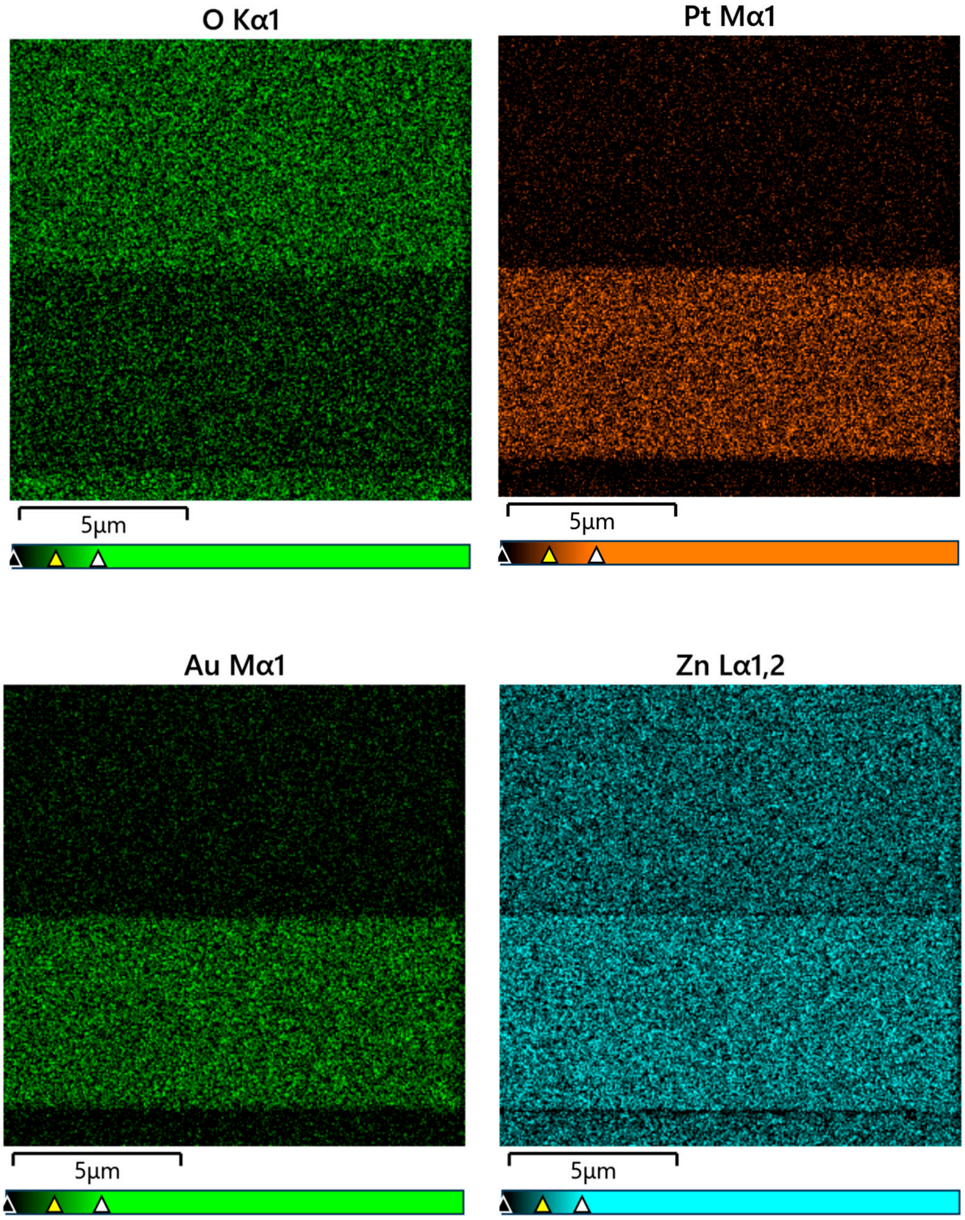

Fig. S4. EDS mapping of ZnO-Au sensing materials.

4. Repeatability and precision of the measurements.

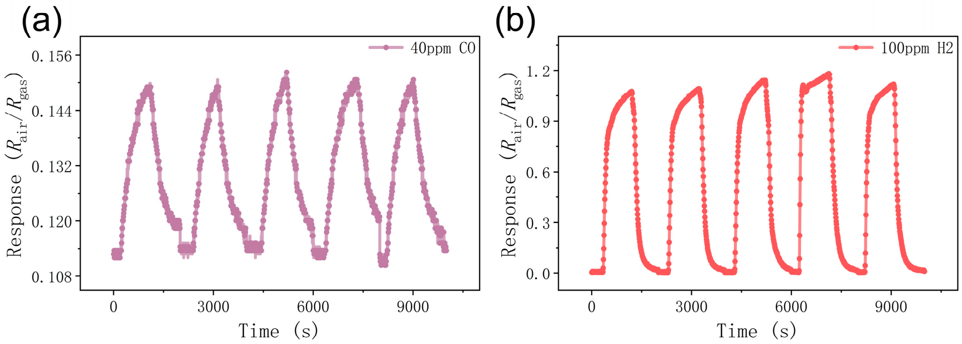

Fig. S5. The repeatability results of the CO and H_2 measurements.

5. The results of different windows under different components in our work.

Table S2. Comparison of the gas classification accuracy of the gas sensor arrays with different components.

Gas sensor array type	Time window	Classification accuracy
Commercial MEMS sensors plus self-made MEMS sensors	5s	94.5%
	10s	98.5%
	15s	99.2%
	20s	99.4%
Only commercial MEMS sensors	5s	89.2%
	10s	94.5%
	15s	96.3%
	20s	96.8%
